# 异基因造血干细胞移植后成人并发腺病毒感染相关肝功能衰竭2例报告并文献复习

**DOI:** 10.3760/cma.j.cn121090-20250804-00361

**Published:** 2026-01

**Authors:** 小进 王, 娜 王, 漾 杨, 阳 曹, 凡凯 孟, 义成 张, 金环 徐

**Affiliations:** 华中科技大学同济医学院附属同济医院血液科，武汉 430030 Department of Hematology, Tongji Hospital, Tongji Medical College, Huazhong University of Science and Technology, Wuhan 430030, China

## Abstract

异基因造血干细胞移植后人腺病毒（HAdV）肝炎虽少见，但病情常进展迅速、死亡率高。本文报道2例淋巴组织增生性疾病患者，接受单倍体造血干细胞移植后发生HAdV感染并死于肝功能衰竭的诊疗过程，提示我们应加强对高危患者HAdV的早期多部位筛查、动态监测并及时干预，以期降低患者死亡风险并改善预后。

## Abstract (en)

Human adenovirus（HAdV）hepatitis is a rare but rapidly progressive and often fatal complication following allogeneic hematopoietic stem cell transplantation. The present study reported the management of two patients with lymphoproliferative disease who developed HAdV infection after haploidentical hematopoietic stem cell transplantation and subsequently died of liver failure. These cases highlight the importance of early multi-site screening, dynamic monitoring, and timely intervention for HAdV in high-risk patients to reduce mortality risk and improve prognosis.

异基因造血干细胞移植（allo-HSCT）作为血液系统恶性肿瘤患者的重要治疗手段，可显著提高患者的生存率及治愈率[1]。人腺病毒（HAdV）感染在接受allo-HSCT的成年患者中是一种罕见且与不良预后相关的严重并发症[2]，发生率为4％～9％[2]–[5]。人类对HAdV普遍易感，在免疫功能正常的人群中，HAdV通常仅引起自限性感染，主要累及胃肠道、呼吸道以及结膜等部位[6]；然而，在免疫功能低下的人群中，尤其是接受allo-HSCT的患者，有时可能导致严重肠胃炎、膀胱炎、肺炎、脑炎、肝炎甚至播散性疾病[7]，其中，allo-HSCT后HAdV肝炎相对少见，但患者一旦进展为急性肝功能衰竭，病情将会进展迅速且死亡率高[8]。本文报告2例因allo-HSCT后HAdV肝炎进展为急性肝功能衰竭而死亡的成人病例并进行文献复习。

## 病例资料

例1，女，33岁，因“间断发热、全身酸痛10余天”于2023年1月入院。既往身体健康，完善相关检查确诊为伯基特淋巴瘤ⅣB期，国际预后指数（IPI）高风险，中枢神经系统、骨髓未累及，移植前疾病达完全缓解。2023年5月9日接受单倍体造血干细胞移植［供者为其胞兄，36岁，人类白细胞抗原（HLA）5/10相合，供受者血型B+供A+］，预处理采用兔抗人胸腺细胞免疫球蛋白（rATG）进行体内T细胞清除，移植后以环孢素A（CsA）预防移植物抗宿主病（GVHD），伐昔洛韦预防病毒激活。移植后每周检测巨细胞病毒（CMV）及EB病毒（EBV）均未见激活，无急性GVHD表现。8月2日［移植后第85天（+85 d）］患者出现口腔黏膜炎，口腔分泌物检测提示单纯疱疹病毒1型（HSV1）感染，先后予喷昔洛韦和膦甲酸钠治疗各2周后HSV1转阴，黏膜炎症状明显改善。8月16日（+99 d）患者开始间断出现发热，伴咳嗽、咳痰，胸部CT提示肺部感染，经抗感染及糖皮质激素治疗后发热控制，但后续多次复查胸部CT，其肺部影像学表现未明显改善。9月10日（+124 d）糖皮质激素减量后患者再次发热，9月11日（+125 d）复查胸部CT示双肺感染较前进展，但因患者血小板持续低下未行纤维支气管镜检查。9月13日（+127 d）外周血病原学二代测序（NGS）检出人类腺病毒C种（HAdV-C），序列数59424，相对丰度99.46％。此外，还检出少量BK病毒、多瘤病毒及HSV1，遂使用利巴韦林（0.3 g，每天2次，静脉滴注）及西多福韦（5 mg/kg，每周1次，静脉滴注）抗病毒治疗。9月26日（+140 d）外周血PCR示HAdV载量为2.16×10^5^拷贝数/ml，10月7日（+151 d）咽拭子检出HAdV及副流感病毒核酸，10月17日（+161 d）外周血HAdV载量为1.42×10^5^拷贝数/ml，继续予以西多福韦（5 mg/kg，每周1次，静脉滴注）抗病毒治疗，但患者仍间断发热、咳嗽、咳痰，未吸氧状态下血氧饱和度偏低（90％～92％）。10月23日（+167 d）输注血小板后行纤维支气管镜检查，支气管肺泡灌洗液NGS示：HAdV-C（序列数106701，相对丰度99.13％）和HAdV-D（序列数3，相对丰度0.02％），虽经多联用药治疗，HAdV载量未见明显下降。患者移植后整个发热期间动态监测肝功能，并常规予以保肝治疗，10月6日（+150 d）起总胆红素升高，停用肝脏毒性药物，完善检查排除嗜肝病毒性肝炎以及GVHD、肝小静脉闭塞病、移植相关血栓性微血管病等移植后肝脏损害的常见并发症，加强保肝及退黄治疗，效果不佳。10月25日（+169 d）总胆红素呈持续性升高（图1A），外周血HAdV载量升至4.21×10^5^拷贝数/ml，腹部CT平扫提示肝内片状稍低密度影（图2），肝功能进行性恶化，自11月3日（+178 d）起间断予以血浆置换、血浆胆红素吸附及血液灌流等血液净化治疗，但丙氨酸氨基转移酶（ALT）、天门冬氨酸氨基转移酶（AST）及总胆红素仍进行性升高，治疗效果差。11月9日（+184 d）患者出现心功能衰竭伴休克，抢救无效死亡。

**图1:**
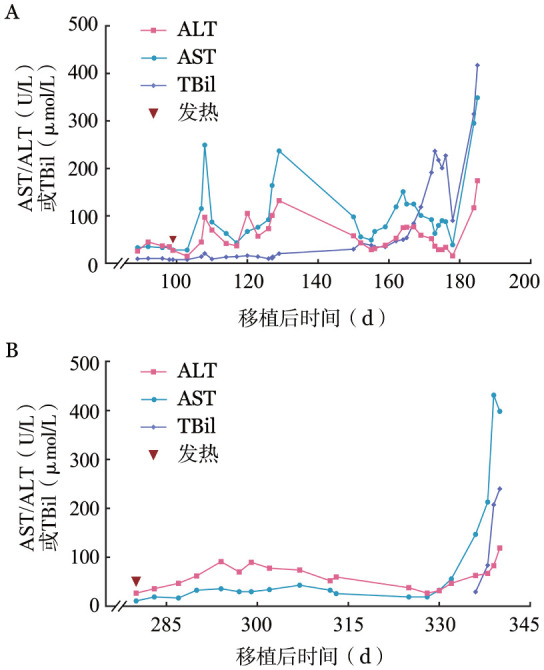
异基因造血干细胞移植后并发腺病毒感染相关肝功能衰竭患者的转氨酶、总胆红素（TBil）的变化情况 **A** 例1；**B** 例2 **注** ALT：丙氨酸氨基转移酶；AST：天门冬氨酸氨基转移酶

**图2:**
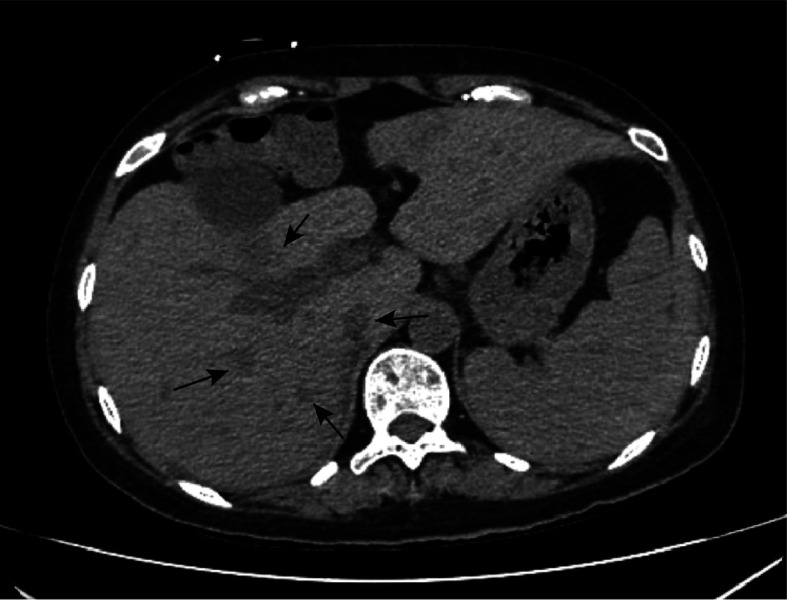
伯基特淋巴瘤患者（例1）单倍体造血干细胞移植后169 d的腹部CT图 **注** 肝脏可见散在片状稍低密度影（箭头所指处）

例2，男，20岁，2022年8月于上海某医院确诊为急性T淋巴细胞白血病（ALL）（高危组），经化疗后疾病达完全缓解。2023年2月26日接受单倍体造血干细胞移植，5月29日（+92 d）行PET-CT提示ALL浸润左侧胸壁及胸膜；骨髓细胞学示骨髓增生减低；流式细胞术检测微小残留病（MRD）为阴性；骨髓完全嵌合。患者髓外复发，行左胸壁病灶放疗（30 Gy/10 Fx），完成9次放疗后至我院就诊，后续经多种方案化疗，效果不佳，为挽救患者生命，于9月11日（+197 d）回输CD7 CAR-T细胞。11月16日（+263 d）复查PET-CT示胸壁及胸膜病灶消失，但出现鼻咽部浸润；骨髓细胞学仍示骨髓增生减低、流式细胞术MRD阴性、嵌合未检测。12月3日（+280 d）患者出现间断发热，次日外周血NGS检出HAdV-C（序列数30918，相对丰度88.74％）。自12月5日（+282 d）起予以西多福韦（5 mg/kg，每周1次，静脉滴注）抗HAdV治疗，3周后鼻咽拭子亦检出HAdV-DNA，加用喷昔洛韦治疗，患者仍反复发热，2024年1月15日（+323 d）外周血NGS示HAdV-C（序列数1036，相对丰度7.40％）；1月22日复查外周血NGS示HAdV-C序列数升至5544（相对丰度19.53％），同时检出细环病毒及分枝横梗霉，继续加强抗感染治疗。患者因既往allo-HSCT及细胞免疫治疗后多次复发、原发病进展，合并重度免疫缺陷，造血重建不良及长期输血依赖，决定行二次挽救移植，2024年1月22日患者接受无关供者造血干细胞移植（骨髓库供者，男，9/10，O+供O+），预处理采用rATG进行体内T细胞清除，使用CsA预防GVHD。1月28日（二次移植+6 d）患者ALT、AST及总胆红素突然进行性升高（图1B），外周血HAdV载量为9.12×10^4^拷贝数/ml，血清铁蛋白>50 000 µg/L，予以血浆置换并加强保肝退黄治疗，但肝功能仍持续恶化。2月2日（二次移植+11 d）清晨患者出现烦躁不安，急查血氨为102 µmol/L，考虑肝功能衰竭合并肝性脑病可能性大，予以血浆置换、血浆胆红素吸附、降血氨、保肝退黄等综合治疗，效果不佳。当日20时14分，患者突发呼吸循环衰竭，抢救无效死亡。

## 讨论及文献复习

HAdV是一类双链DNA病毒，目前已鉴定出A到G共7个种群，涵盖超过100种基因型[9]。HAdV感染可累及全身多系统，常见受累部位包括胃肠道、呼吸道及结膜等[8],[10]。国外报道显示，接受allo-HSCT患者的HAdV感染发生率为0～20％或更高[6]，其中成人发生率低于儿童（4％～9％对15％～32％）[2]–[5]；相比之下，国内数据较为有限，周斐等[11]报道成人allo-HSCT后HAdV感染发生率约0.78％；龙彦等[12]报道其总体发生率为3.4％，同样提示成人低于儿童（3.3％对3.8％）。根据欧洲白血病感染会议（ECIL）指南[13]，HAdV感染的危险因素包括儿童接受去T细胞处理或无关供者移植、成人接受单倍体造血干细胞移植或阿仑单抗治疗、无关脐血移植、严重（Ⅲ～Ⅳ级）GVHD以及严重淋巴细胞减少症（淋巴细胞计数<0.2×10^9^/L）等。此外，淋巴组织增生性疾病（如急/慢性淋巴细胞白血病、非霍奇金淋巴瘤和霍奇金淋巴瘤等）及严重免疫缺陷状态亦属危险因素[2]。本文2例患者均罹患淋巴组织增生性疾病、接受单倍体造血干细胞移植并经历包含rATG的预处理及CsA预防GVHD，例2因复发接受二次移植，二者均处于严重免疫缺陷状态，符合上述危险因素特征。

HAdV肝炎在allo-HSCT后相对少见，主要与HAdV-C（尤其是C5型）相关。这种病毒对肝细胞具有较高亲和力，其六邻体蛋白可与凝血因子Ⅹ等结合，介导病毒进入肝细胞，进入肝细胞的病毒被库普弗细胞大量捕获，以触发白细胞介素-1α、肿瘤坏死因子-α等炎性因子大量释放，进而激活NK细胞和CD8^+^T细胞，最终通过免疫损伤导致肝细胞广泛坏死[14]，从而进展为肝炎甚至肝功能衰竭。HAdV肝炎早期表现不典型，具体为无症状感染或仅表现出发热、腹泻等非特异性症状；实验室检查可见转氨酶升高和全血细胞减少[6]；外周血检出HAdV-DNA、腹部增强CT显示肝脏低密度灶，或发病前2周γ-谷氨酰转移酶升高（>100 IU/L）等有助于早期识别[15]，而确诊仍依赖于肝组织活检。鉴于HAdV组织嗜性的不同，联合多部位筛查（如外周血、粪便、鼻咽拭子、支气管肺泡灌洗液、脑脊液或病理组织）有助于避免漏诊。常用检测手段包括病毒分离培养、免疫学与血清学检测、PCR、宏基因组高通量测序技术和组织病理学检查[13],[16]，临床常用PCR检测HAdV-DNA[6]。ECIL建议对高危患者每周进行至少1次外周血HAdV检测，直到其完成充分的免疫重建[13]。

值得注意的是，本文例2在CAR-T细胞治疗后再移植过程中出现HAdV再激活，提示多重免疫因素在病毒控制中的关键作用。该患者在CAR-T细胞治疗前已有移植史，处于基础免疫抑制状态；首次CAR-T细胞输注后84 d出现发热，外周血NGS提示HAdV感染，其发生可能与淋巴细胞清除化疗所致黏膜屏障损伤和全血细胞减少以及CAR-T细胞治疗引起的T、B细胞耗竭和低丙种球蛋白血症等因素有关[17]–[18]。尽管西多福韦治疗曾使病毒载量短暂下降，但二次移植前的rATG预处理进一步强化了T细胞清除，最终导致患者移植后HAdV病毒载量再次上升。该例提示，对于存在CAR-T细胞治疗、二次移植等多重免疫抑制因素叠加的患者，动态监测HAdV、早期识别并采取抢先干预具有重要临床意义。

目前HAdV感染尚无标准治疗方案，主要治疗策略包括免疫抑制剂减量或停用、抗病毒药物治疗及过继性T细胞免疫治疗等[13]。然而，现有疗法总体疗效有限，尤其对于播散性感染患者，死亡率为60％～80％[19]，提示早期识别并及时干预至关重要。因此，对allo-HSCT后长期不明原因发热的患者，应监测外周血HAdV病毒载量；若检测阳性且HAdV-DNA>1×10^3^拷贝数/ml[20]，或连续2次检测病毒载量上升10倍及以上，应启动抢先治疗[21]。西多福韦是抢先治疗的主要药物，可抑制病毒复制并预防播散，在病毒学应答良好的患者中，首次给药后3～10 d可见病毒载量下降，但病毒清除时间取决于宿主T细胞重建及HAdV特异性T细胞的形成，该过程通常需数周至数月[22]–[23]；在体温控制方面，Moon等[24]研究显示，西多福韦组重症腺病毒肺炎患者的退热时间较非西多福韦组平均缩短2.1 d，但差异无统计学意义。西多福韦对晚期播散性感染的疗效尚存争议[25]–[26]，且其具有较强的肾毒性，目前国内主要限于临床研究或同情使用。而其他常用抗病毒药物，如利巴韦林、更昔洛韦及磷甲酸钠，在临床应用中均未表现出明确的抗HAdV活性[6],[14]。本文2例患者均于发热后检出HAdV，虽经西多福韦、利巴韦林等多种抗病毒药物联合治疗，其体温、病毒载量及肝功能均未获得持续改善，病情持续进展，反映出重症HAdV感染的治疗难度及晚期干预的局限性。

HAdV肝炎作为allo-HSCT后相对少见的并发症，临床表现异质性高且缺乏特异性，早期诊断困难，一旦进展至肝功能衰竭，病情常进展迅速，死亡率高。因此，临床应对高危患者保持高度警惕，加强HAdV的早期筛查、动态监测与及时干预，以降低死亡风险并改善预后。

## References

[1] Díaz-Lagares C, Fox L, García-Roche A (2021). Sequential organ failure assessment score and the need for organ support predict mortality in allogeneic stem cell transplant patients admitted to the intensive care unit[J]. Transplant Cell Ther.

[2] Balletto E, Ponzano M, Raiola AM (2024). Adenovirus infection in adult patients undergoing allogeneic hematopoietic stem cell transplant: Incidence, clinical management, and outcome[J]. Transpl Infect Dis.

[3] Sedláček P, Petterson T, Robin M (2019). Incidence of adenovirus infection in hematopoietic stem cell transplantation recipients: findings from the AdVance study[J]. Biol Blood Marrow Transplant.

[4] Papanicolaou GA, Dvorak CC, Dadwal S (2020). Practice patterns and incidence of adenovirus infection in allogeneic hematopoietic cell transplant recipients: Multicenter survey of transplant centers in the United States[J]. Transpl Infect Dis.

[5] Cesaro S, Berger M, Tridello G (2019). A survey on incidence and management of adenovirus infection after allogeneic HSCT[J]. Bone Marrow Transplant.

[6] Cesaro S (2023). Adenovirus infection in allogeneic hematopoietic cell transplantation[J]. Transpl Infect Dis.

[7] Tomoda T, Nishimura A, Kamiya T (2024). Immune reconstitution and cidofovir administration rescue human adenovirus hepatitis after allogeneic hematopoietic cell transplantation[J]. Transpl Immunol.

[8] Onda Y, Kanda J, Sakamoto S (2021). Detection of adenovirus hepatitis and acute liver failure in allogeneic hematopoietic stem cell transplant patients[J]. Transpl Infect Dis.

[9] Luo H, Zhou Q, Feng J (2024). Global prevalence of preexisting antibodies against human adenoviruses, surveyed from 1962 to 2021[J]. Intervirology.

[10] Shieh WJ (2022). Human adenovirus infections in pediatric population-An update on clinico-pathologic correlation[J]. Biomed J.

[11] 周 斐, 陈 苏宁, 吴 德沛 (2023). 异基因造血干细胞移植后腺病毒感染26例诊治分析[J]. 中华血液学杂志.

[12] 龙 彦, 孙 媛媛, 刘 畅 (2017). 异基因造血干细胞移植后患者腺病毒感染监测及临床意义[J]. 中华检验医学杂志.

[13] Matthes-Martin S, Feuchtinger T, Shaw PJ (2012). European guidelines for diagnosis and treatment of adenovirus infection in leukemia and stem cell transplantation: summary of ECIL-4 (2011)[J]. Transpl Infect Dis.

[14] Zheng N, Wang Y, Rong H (2022). Human adenovirus associated hepatic injury[J]. Front Public Health.

[15] Kawashima N, Muramatsu H, Okuno Y (2015). Fulminant adenovirus hepatitis after hematopoietic stem cell transplant: retrospective real-time PCR analysis for adenovirus DNA in two cases[J]. J Infect Chemother.

[16] 董 静肖, 赵 秀英 (2023). 人腺病毒感染现状及分型检测技术的研究进展[J]. 中华检验医学杂志.

[17] Brudno JN, Kochenderfer JN (2016). Toxicities of chimeric antigen receptor T cells: recognition and management[J]. Blood.

[18] Riedel A, Phely L, Hug S (2025). Immunological consequences of CAR T-cell therapy: an analysis of infectious complications and immune reconstitution[J]. Blood Adv.

[19] Matthes-Martin S, Boztug H, Lion T (2013). Diagnosis and treatment of adenovirus infection in immunocompromised patients[J]. Expert Rev Anti Infect Ther.

[20] Hiwarkar P, Kosulin K, Cesaro S (2018). Management of adenovirus infection in patients after haematopoietic stem cell transplantation: State-of-the-art and real-life current approach: A position statement on behalf of the Infectious Diseases Working Party of the European Society of Blood and Marrow Transplantation[J]. Rev Med Virol.

[21] Grimley MS, Chemaly RF, Englund JA (2017). Brincidofovir for asymptomatic adenovirus viremia in pediatric and adult allogeneic hematopoietic cell transplant recipients: a randomized placebo-controlled phase II trial[J]. Biol Blood Marrow Transplant.

[22] Anderson EJ, Guzman-Cottrill JA, Kletzel M (2008). High-risk adenovirus-infected pediatric allogeneic hematopoietic progenitor cell transplant recipients and preemptive cidofovir therapy[J]. Pediatr Transplant.

[23] Leruez-Ville M, Minard V, Lacaille F (2004). Real-time blood plasma polymerase chain reaction for management of disseminated adenovirus infection[J]. Clin Infect Dis.

[24] Moon SM, Choe J, Na SJ (2021). Comparative study on the effect of cidofovir treatment for severe adenovirus pneumonia[J]. J Intensive Care Med.

[25] Thomas SJ, Young RT, Steinbach WJ (2021). Risks and outcomes of adenovirus disease in pediatric hematopoietic stem cell transplant recipients-comparison of current antiviral treatment options[J]. Transpl Infect Dis.

[26] Chandorkar A, Anderson AD, Morris MI (2021). Viral kinetics and outcomes of adenovirus viremia following allogeneic hematopoietic cell transplantation[J]. Clin Transplant.

